# Increased NETosis in Patients With Heart Failure Is Associated With Macrophage Activation Towards a Proinflammatory Phenotype

**DOI:** 10.1111/jcmm.71306

**Published:** 2026-09-11

**Authors:** Sawa Kostin, Hector A. Cabrera‐Fuentes, Manfred Richter, Florian Krizanic, Oliver Ritter, William A. Boisvert, Klaus T. Preissner, Theodoros Kelesidis, Gerasimos Siasos, Nikolaos Pagonas

**Affiliations:** ^1^ Faculty of Health Sciences Brandenburg Brandenburg Medical School Theodor Fontane Neuruppin Germany; ^2^ División de Estudios de Posgrado e Investigación Tecnológico Nacional de México/Instituto Tecnológico de Tijuana Tijuana Mexico; ^3^ R&D Office, Vice Presidency Scientific Research & Innovation, Imam Abdulrahman Bin Faisal University (IAU) Dammam Saudi Arabia; ^4^ Department of Cardiac Surgery Kerckhoff‐Clinic Bad Nauheim Germany; ^5^ Department of Internal Medicine and Cardiology Medical School Theodor Fontane, University Hospital Ruppin‐Brandenburg Neuruppin Germany; ^6^ Medical School Theodor Fontane, University Medical Center Brandenburg an der Havel Brandenburg an der Havel Germany; ^7^ Center for Cardiovascular Research, John A. Burns School of Medicine University of Hawaii Honolulu Hawaii USA; ^8^ Department of Cardiology, Kerckoff Heart Research Institute Justus‐Liebig‐University Giessen Germany; ^9^ Southwestern Medical Center, University of Texas Dallas Texas USA; ^10^ 3rd Department of Cardiology ‘Sotiria’ Chest Disease Hospital, Medical School, National and Kapodistrian University of Athens Athens Greece

**Keywords:** heart failure, inflammation, macrophages, NETosis, neutrophils

## Abstract

In this study, we tested the hypothesis that there is an interaction between neutrophils (N) undergoing NETosis and the activation of macrophages (Mf) in the myocardium of patients with heart failure (HF). Ventricular biopsies were examined from patients with HF due to dilated (DCM, *n* = 7), ischemic (ICM, *n* = 7), or inflammatory (InfCM, *n* = 7) cardiomyopathy, as well as from 5 control patients. Quantification of CD66b^+^/citrullinated histone 3^+^/myeloperoxidase^+^ neutrophils (N) revealed that 49.4% [IQR, 45–52] of N developed NETosis in InfCM, 45.3% [39–46] in ICM, 41.5% [37–45] in DCM, and 4% [3.8–4.2] in the control group. Quantification of the proportion of CD66b^+^/TNF‐α^+^ Mf1 in infCM yielded a value of 78.5% [70–85], which differed significantly (*p* < 0.05) from that in DCM (53.1% [48.4–54.4]) or ICM (54.5% [51–60]). The proportion of Mf1 in the control group was 3% [1.6–5.5] and differed significantly (*p* < 0.01) from all HF groups. The proportion of CD66b^+^/CD206^+^ Mf2 was 20.9% [17.9–27.7] in DCM, 21.1% [17.8–22.8] in ICM, and 23.2% [21.2–25.1] in infCM. The proportion of Mf2 in the control group was 6.5% [4.8–7.6] and differed significantly (*p* < 0.01) from all HF groups. Correlation analysis revealed a statistically significant positive correlation (*r* = +0.79, *p* < 0.01) between Mf1 and the proportion of NETosis. In contrast, there was no significant correlation between Mf2 and the proportion of NETosis (*r* = −0.12, *p* = 0.28). It is concluded that the accumulation of N and Mf cells is a typical feature of chronic myocardial inflammation in HF, regardless of its aetiology. An increased number of N cells undergoing NETosis HF is associated with the activation of myocardial Mf cells and causes these cells to adopt a proinflammatory, Mf1‐like phenotype.

## 1 Introduction

Heart failure (HF) is pathological structural myocardial remodelling including cardiomyocyte hypertrophy, fibrosis, cardiomyocyte cell death and fibrosis. Chronic, low‐grade inflammation is also one of the major factors implicated in the development and progression of HF [1, 2, 3]. Polymorphonuclear neutrophils are terminally differentiated cells and are the most abundant subset of leukocytes and the major constituents of the innate immune system. These short‐lived cells are potent triggers of inflammatory responses, and their role in the context of cardioprotection, ischemia/reperfusion injury, myocardial infarction (MI) and post‐MI remodelling has been studied extensively and reviewed in detail [4, 5, 6, 7, 8, 9, 10, 11, 12]. Neutrophils also play a significant role in the development and progression of HF, as they cause tissue damage and pathological cardiac remodelling [6, 13, 14]. In addition, neutrophils have been shown to play an important role in the pathogenesis of HF regardless of aetiology, disease severity, and all‐cause mortality [15].

Neutrophils are key components of the innate immune system and can undergo an activation process consisting of the formation of the extracellular reticular web‐like network known as NET (Neutrophil Extracellular Trap). NETs were originally recognised as a mechanism for trapping and killing microbes, boosting the immune response to fight infections [16, 17]. Furthermore, this unique process of NET formation was associated with the death of neutrophil cells and was therefore called NETosis [18]. However, NET formation can either occur with preservation of neutrophilic functions (vital NETosis) or with neutrophil cell death (suicidal or lytic NETosis) [18, 19]. The major differences between vital NETosis and suicidal NETosis are the sources of stimuli and the pathways involved in NET formation [20, 21]. It has been demonstrated that vital NETosis is induced by pathogen‐specific molecular patterns (PAMPs), whereas suicidal NETosis is induced by tissue‐related damage–associated molecular patterns (DAMPs) to initiate inflammation and promote tissue repair [18, 22].

A number of cellular and molecular events are involved in NET formation and NETosis, including the decondensation of heterochromatin and the unfolding of chromatin by citrullination of histones by the protein peptidyl‐arginine deiminase 4 [23]. Another event involved in NET formation is neutrophil degranulation leading to the translocation of neutrophil elastase (NE) and myeloperoxidase (MPO) from the granules to the nucleus and the cleavage of histones to promote chromatin decondensation [24, 25]. The latter, together with the destruction of the nuclear envelope, leads to the release of chromatin into the cytoplasm. In the final stage of NETosis, DNA chains, citrullinated histones and the granular proteases NE and MPO are released into the extracellular space due to perforation of the neutrophil plasma membrane, resulting in NETs [22]. In the field of cardiovascular diseases, NETosis has been shown to play an important role in atherosclerosis and thrombo‐inflammation. Although the role of NETosis has been suggested as an important component of chronic myocardial inflammation in clinical and experimental HF as well, this needs further investigation.

Macrophages are among the most active cell types involved in the immune response of the heart, and contribute to both the development and resolution of inflammation [26, 27, 28, 29]. These cells exhibit a high degree of plasticity, which enables them to adapt their functional phenotype to the respective environment. Macrophages can basically be divided into two phenotypically distinct populations with different gene expression profiles and functions; namely classically activated M1‐like macrophages and alternatively activated M2‐like macrophages [30, 31, 32, 33, 34, 35, 36, 37, 38, 39, 40, 41]. Although this is a highly simplified view of macrophage behaviour [42, 43], general guidelines suggest that the M1 and M2 nomenclature can be useful for a basic characterisation of macrophage phenotypes [44].

Macrophages are one of the most active cell types participating also in the cardiac remodelling events that occur during the inflammatory, proliferative and reparative phases after a MI. Macrophage polarisation in MI has been well researched from the acute stage to scar formation and post‐remodelling after MI [29, 45, 46, 47]. It has been reported that macrophages show a highly regulated, time‐dependent appearance of classical M1‐like and alternative M2‐like activation during different stages following MI [29, 47, 48]. Whether macrophage polarisation occurs in patients with HF is still speculative and largely unknown [3, 27, 49, 50]. Thus, although the role of macrophage activation and polarisation in inflammation and healing after acute MI is relatively well defined, less is known about these processes in the context of chronic HF, and this remains to be clarified.

Several studies reported a reciprocal interplay between macrophage polarisation and NETosis [51, 52, 53, 54, 55]. While some studies demonstrated that neutrophils undergoing NETosis promote pro‐inflammatory M1‐like macrophages [55], other studies showed that NETosis induces polarisation of macrophages towards an M2‐like phenotype [8, 53, 56]. In the context of HF, virtually nothing is known about the interplay between the phenotype of macrophages and NETosis.

From this point of view, the present study set out to further investigate NETosis and to characterise the phenotype of myocardial macrophages in HF. Furthermore, we tested the hypothesis that there is a correlation between the phenotypic changes of macrophages and NETosis in patients with HF.

## 2 Material and Methods

### 2.1 Patients

Left ventricular (LV) samples were collected from the explanted hearts of patients with end‐stage HF undergoing orthotopic heart transplantation. Patients either had normal coronary arteries with previously proven myocarditis and subsequent development of chronic HF (inflammatory CMP, infCM, *n* = 7) or without previously histologically proven myocarditis (idiopathic dilated CMP, DCM, *n* = 7), or had severe coronary artery disease (ischemic CMP, ICM, *n* = 7) with a history of previous myocardial MI. Diagnosis of ICM was based on clinical history, Doppler echocardiography and cardiac catheterisation data. Patients were functionally classified according to criteria of the NYHA and were listed for heart transplantation according to the guidelines for organ transplantation of the German Medical Association. As previously described [57], from the patients with ICM, only the regions distant from MI (remote regions) were investigated. The HF groups were matched for age, NYHA class, medications and comorbidities (Table S1). LV samples from five patients with aortic stenosis but with preserved left ventricular function (> 60%)—served as control tissue. The control samples showed no signs of myocardial damage, inflammation, or fibrosis. All studies were approved by the ethical committee of the Landesaerztekammer Hessen (project number FF 8/2011 and FF56/2012), and all patients gave written consent.

### 2.2 Tissue Sampling

Ventricular myocardial tissue biopsies were washed with a cold Krebs–Henseleit solution augmented with 0.1% adenosine and 0.5% albumin. Tissue samples were immediately frozen for immunohistochemistry, Western blot, and RT‐qPCR and stored at −80°C until use.

### 2.3 Immunohistochemistry

The tissue samples were mounted in Tissue‐TeK O.C.T.TM (Sakura) and 5 μm cryosections were prepared using a Leica CM3050S cryotome. Tissue sections were fixed in 4% freshly prepared paraformaldehyde for 10 min. After washing in PBS, sections were incubated for 30 min with 2% bovine serum albumin and 0.4 mg/L glycine in 0.1% TritonX‐100 in PBS, followed by subsequent blocking with 2% normal non‐immune goat (ab7481, Abcam) and 2% donkey serum (ab7475, Abcam) in PBS for one hour at room temperature. Primary antibodies against CD66b (1:50, Ab197678, Abcam), CEACAM8/CD66b (1:100, NBP2‐80664, G10F5, Novus Biological), MPO (1:200, AF 3667, R&D Systems) and citH3 (1:100, ab5103, Abcam) were used to identify neutrophils and detect NETosis. Primary antibodies against CD68 (1:150, ab222914, Abcam), CD206, arginase‐1 (1:25, D4E3M, Cell Signalling), TNFα (1:200, TNFA, ab220210, Abcam) and IL‐6 (1:100, 8H12, ThermoFisher Scientific) were used to identify macrophages and characterise their phenotypes. Secondary antibodies were donkey anti‐mouse IgG‐conjugated with Cy3 (1:200, 715‐165‐150, Dianova), AlexaFluor594 (1:200, ab150116, Abcam), goat anti‐mouse, μ‐chain specific IgM conjugated with Cy2 (1:100, AB_2338754, Jacksohn ImmunoResearch), Cy 3 donkey anti‐rabbit IgG (1:300, 711‐166‐152, Dianova), AlexaFluor594 (1:200, A‐11012. Invitrogen), Cy3 donkey anti‐goat IgG (1:300, 705‐165‐147, Dianova), and were incubated for one hour at room temperature. For multiple immunolabelling, each primary antibody and its detection systems were tested separately for optimal antibody dilutions. The double and triple immunolabelling was performed using a sequential incubation with the primary antibodies. The nuclei were stained with 1 μg/mL 4′,6‐diamidino‐2‐phenylindole (DAPI, Molecular Probes). F‐actin was fluorescently stained using Alexa647‐conjugated phalloidin (Molecular Probes). The sections were embedded in Mowiol and then covered with a cover glass.

### 2.4 Cell Quantification

For cell quantification, cryosections from at least two different tissue blocks in each case were used. The image acquisition for quantifications was done using a ×40 Planapo objective (Leica) and a Leica (Leitz DMRB) fluorescent microscope equipped with a Leica DC380 digital camera. The quantification of neutrophils undergoing NETosis was performed blindly, with only CD66b‐positive cells displayed on the screen for the calculation of the total number of neutrophils. The images displaying citH3 and MPO were captured from the same field of view, and the percentage of neutrophils undergoing NETosis was calculated as the proportion of cells in which CD66b colocalised with either citH3 or MPO, or with both. Similarly, quantification of the macrophage phenotype was performed blindly, with only CD68‐positive cells displayed on the screen to calculate the total number of macrophages. Images of TNF‐α and CD206 were captured from the same image section, and the percentage of macrophages with the macrophage phenotype was calculated. Cells with colocalised CD68 and TNFα were quantified as M1‐like macrophages and cells with CD68 and CD206 were quantified as M2‐like macrophages. The cells were quantified blindly, without knowledge of the groups examined. For each patient, at least 10 randomly selected fields of view were analysed using Image J program. The number of cells were calculated per 1 mm^2^ surface area as described [58, 59].

### 2.5 Confocal Microscopy

Tissue sections were examined by laser scanning confocal microscopy (Leica SP2 and Leica SP5). Series of confocal optical sections were taken using a Leica Planapo ×40/1.00 or ×63/1.32 objective lens. To avoid or minimise spectral interference between different fluorochromes, confocal fluorescence images with 4 and 5 channels were acquired using sequential single‐channel scans. The images were overlaid and the quality improved using Imaris multi‐channel image processing software (Bitplane, Zurich, Switzerland) to achieve a high signal‐to‐noise ratio.

### 2.6 Western Blot Analysis

Tissue samples and cells were processed for Western blot analysis as previously described [60]. In brief, frozen tissues were homogenised in RIPA buffer (containing 20 mmol/L Tris–HCl at pH 7.4, 100 mmol/L NaCl, 5 mmol/L thylene‐diamine tetra‐acetic acid, 1% Triton X‐100, 10% glycerol, 0.1% sodium dodecylsulfate, 1% deoxycholate, 50 mmol/L NaF, 10 mmol/L Na4P2O7, 1 mmol/L Na3VO4, 1 mmol/L phenylmethylsulfonylfluoride) and mammalian protease inhibitor cocktail (Sigma) at pH 7.4 and centrifuged at 2000× *g* at 4°C for 10 min. Tissue extracts were loaded onto 12% polyacrylamide gel and separated under reducing conditions. Proteins were electro‐transferred onto nitrocellulose membrane (Invitrogen) and blocked with 5% non‐fat dry milk in Tris‐buffered saline Tween‐20 (TBST) at 4°C. After washing with TBST, proteins were exposed overnight at 4°C to against citH3 antibodies (1.1000, Arg17, E4O3F, Cell Signalling) diluted in TBS with 5% powdered milk. Bound antibodies were detected by horseradish peroxidase‐conjugated anti‐rabbit IgG (HAF008, R&D Systems) and Pierce Fast Western Blot Kits, SuperSignal West Femto (Fisher Scientific) detection system and exposed to X‐ray film. Quantification of immunoblots was done by scanning on a STORM 860 (Amersham, Pharmacia Biotech) using ImageQuant software as described [61]. The immunoblotting values for citH3 were normalised per β‐actin (A5441, Sigma‐Aldrich) and the control values were set at 100%.

### 2.7 RT‐qPCR

Total RNA was extracted from frozen human tissue samples (approximately 30 mg) using the RNeasy Mini Kit (Qiagen). The concentration and quality of the isolated RNA were assessed using a Nanodrop 2000 spectrophotometer (PEQLAB Biotechnologie GmbH, Erlangen, Germany). For cDNA synthesis, 200 ng of total RNA was reverse transcribed using the High‐Capacity cDNA Reverse Transcriptase Kit (Applied Biosystems). The following primers were used: MPO (OriGene, catalogue number HP200232, forward sequence: GAGCAGGACAAATACCGCACCA, reverse sequence: AGAGAAGCCGTCCTCATA CTCC), and GAPDH (OriGene, catalogue number HP205798, forward sequence: GTCTCCTCTGACTTCAACAGCG, reverse sequence: ACCACCCTGTTGCTGTAG CCAA). RT‐PCR was performed using the SYBR Green RT‐PCR Kit (Invitrogen) and a StepOne Plus Real‐Time PCR System (Applied Biosystems) according to the manufacturer's instructions. The thermal profile consisted of 2 min at 95°C of activation, followed by 40 PCR cycles of 15 s at 95°C and 20 s at 60°C of annealing. The melting curve was obtained after the last cycle. The assays were performed in triplicate. Gene expression was normalised to GAPDH using the ΔΔCT comparison method and expressed as a fold change compared to the control group.

### 2.8 Statistical Analysis

Statistical analyses were conducted using GraphPad Prism version 10.6.0 for macOS (GraphPad Software, La Jolla, CA, USA; www.graphpad.com). The data distribution was assessed using the Shapiro–Wilk and Brown‐Forsythe tests. Data are presented as a median with interquartile range (Q1–Q3). Statistical analysis was performed using the Kruskal‐Wallis test with the Dunn's correction for multiple comparisons. Correlation between variables was analysed by Spearman's correlation coefficient. A *p* value < 0.05 was considered a significant difference.

## 3 Results

### 3.1 Neutrophil Number and NETosis Are Drastically Increased in HF

**FIGURE 1:**
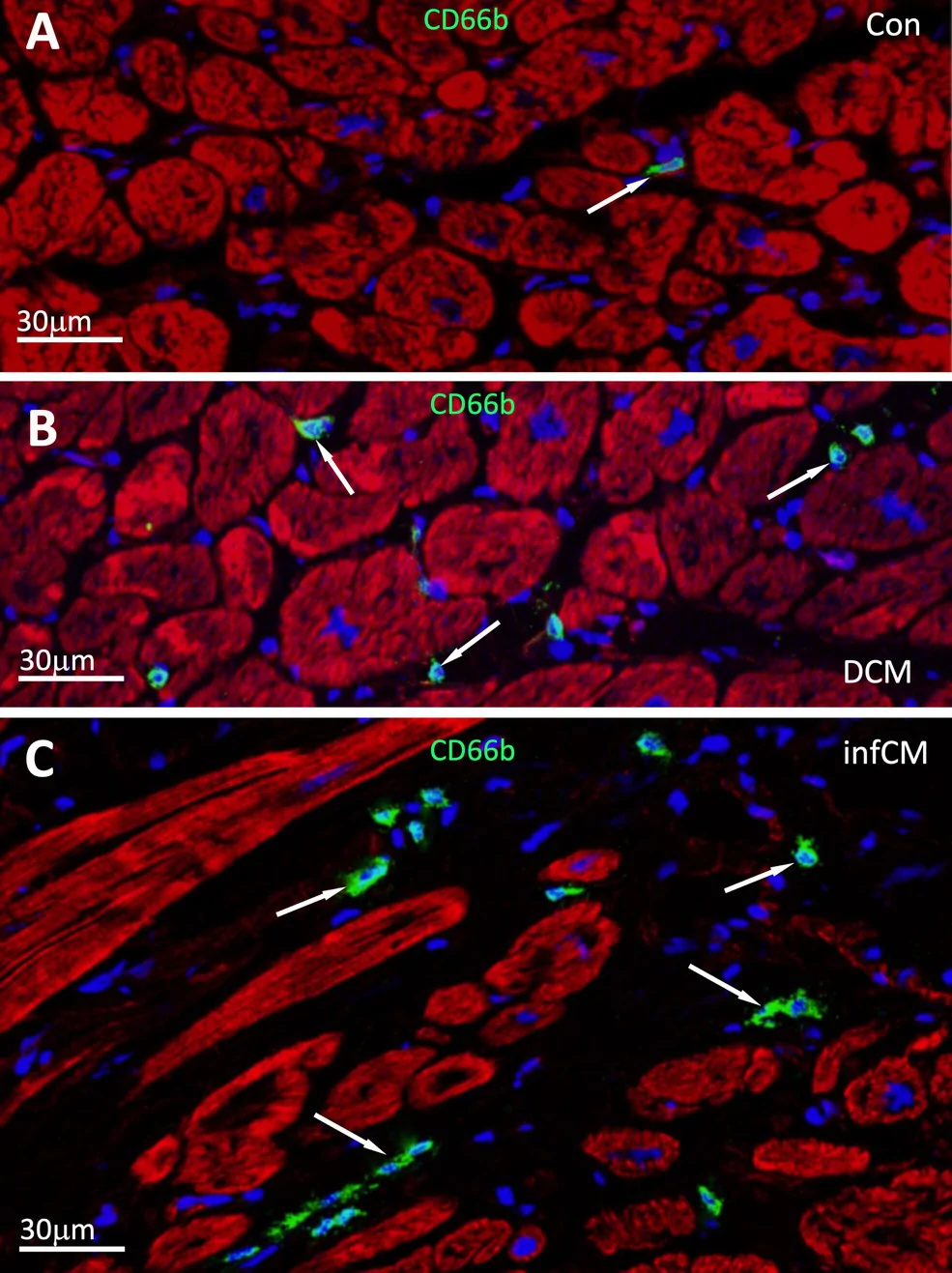
Neutrophils are increased in HF patients. Representative confocal micrographs of CD66b‐positive cells (green, white arrows) in a control (A), DCM (B) and in a patient with infCM (C). Notice a remarkable increase in CD66b‐positive cells in HF patients. Nuclei are shown in blue after staining with DAPI, and F‐Actin is shown in red after labelling with Alexa647‐phalloidin.

To detect the myocardial neutrophils, immunolabelling for CD66b was used. Representative micrographs of CD66b in the examined groups are presented in Figure 1A–C and show that these cells are more numerous in the HF groups than in control patients.

Quantification of CD66b‐positive cells showed a median of 2.48 [1.8–3.44] of neutrophils per 1 mm^2^ of myocardial surface area in the control group, which differed significantly (*p* < 0.01) from that observed in DCM (11.4 [10.5–13.6 cells]), ICM (13.9 [12.3–15.5 cells]), and infCM (15.5 [15.3–16.6 cells]).

**FIGURE 2:**
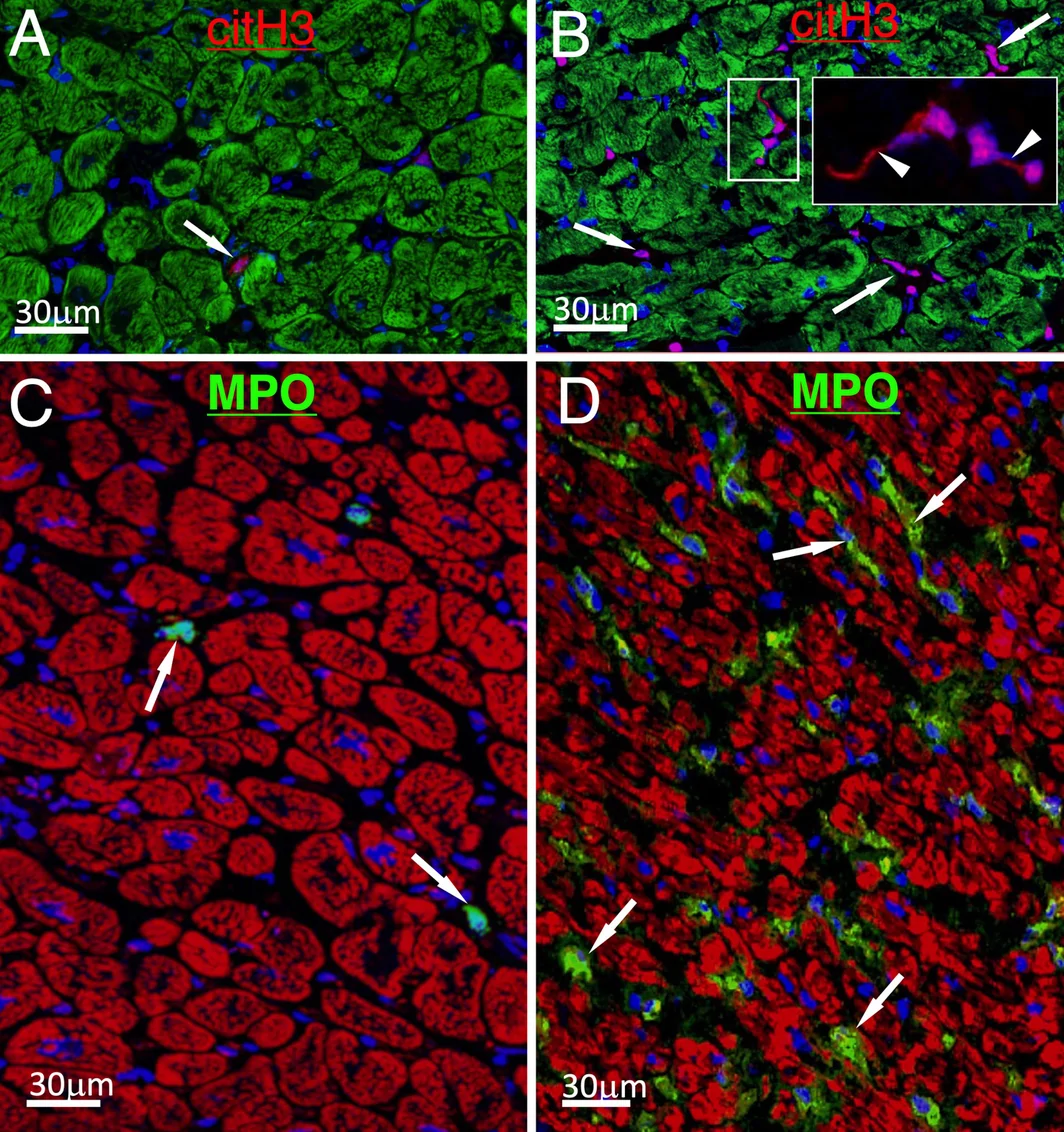
NETosis is increased in HF. Typical immunofluorescent examples of citH3‐positive cells (arrows) in a control patient (A) and in a patient with ICM (B). The insert in (B) is a higher magnification of the boxed region and shows NETS (arrowheads), which are positive for citH3 and DAPI. F‐actin is shown in green and nuclei in blue. MPO‐positive cells in a control (C) and in a patient with infCM (D). Notice a marked increase of the MPO‐positive cells in the latter patient. F‐actin is shown in red and nuclei in blue.

To detect cells undergoing NETosis, we used immune labeling for citrullinated histone 3 (citH3) and myeloperoxidase (MPO). Figure 2A,B as representative confocal images of citH3 labeling demonstrate increased number of citH3‐positive neutrophils in the HF groups as compared to control. It is notable that the myocardium of the HF groups displayed very thin and long (range 5–50 μm) NET structures that are positive for DNA and citH3 (Figure 2B, insert) whereas these structures were not present in the control tissues. The increase in citH3 in HF groups was confirmed by Western blot analysis (Figure S1A,B). The results indicate that the mean citH3 protein level in patients with infCM was 4.8‐fold higher compared to control (*p* < 0.01). In patients with ICM and DCM, citH3‐levels were 4.4‐fold (*p* < 0.01) and 2.7‐fold (*p* < 0.01) higher, respectively, than in control. The citH3 expression in DCM patients was significantly different from that in patients with ICM (*p* < 0.01) and infCM (*p* < 0.01).

Immunolabelling for MPO demonstrated a more pronounced appearance in HF patients compared to controls (Figure 2C,D). These results were confirmed by RT‐qPCR analysis (Figure S1C), which showed that MPO mRNA was 3–4 times higher in the HF groups compared to the control group (*p* < 0.01).

**FIGURE 3:**
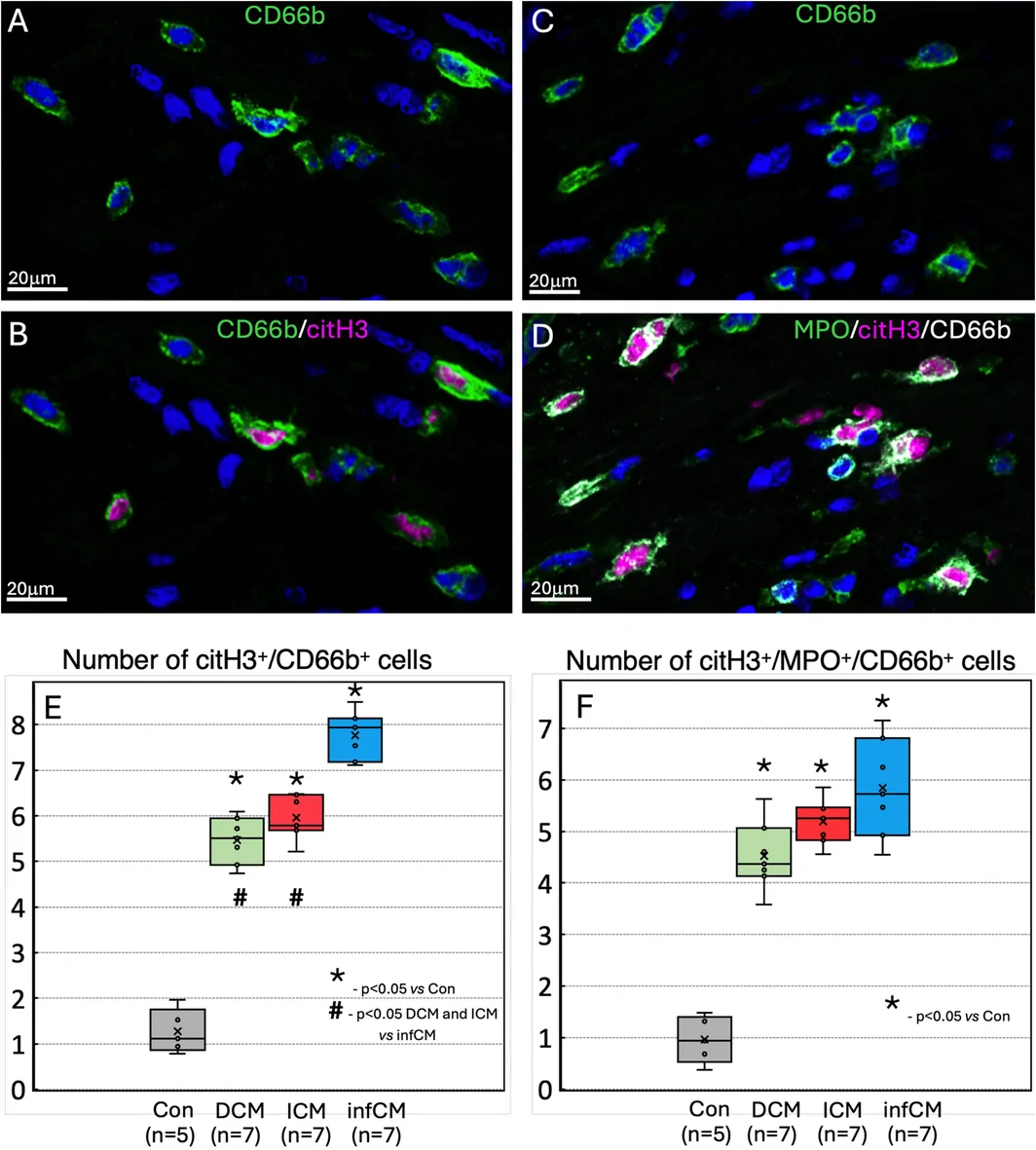
Representative confocal images of CD66b/citH3− (A, B) and CD66b/MPO/citH3‐positive cells (C, D), as well as quantifications of these cells in the groups studied (E, F).

For quantitative analysis of neutrophils undergoing NETosis, tissue sections were double‐stained with CD66b and citH3 (Figure 3A,B) or triple‐stained with CD66b, citH3, and MPO (Figure 3C,D). Quantification of citH3/CD66b‐positive cells revealed that the median value of 7.94 [7.36–8.05] of these cells per 1 mm^2^ of myocardial surface area in patients with infCM differed significantly (*p* < 0.05) from that in patients with DCM (5.51 [5.1–5.84] cells) and ICM (5.79 [5.7–6.38] cells). The medians of citH3/CD66b‐positive cells in all HF groups differed significantly (*p* < 0.01) from the median of 1.12 [0.95–1.53] cells in the control group (Figure 3E). Quantification of MPO/citH3/CD66b revealed a 4.6‐ to 6.1‐fold increase in these cells in HF groups as compared to control (Figure 3F). There were no significant differences between the HF groups in this parameter.

Taken together, these data indicate an increase in neutrophil accumulation with a concomitant increase in neutrophils undergoing NETosis in the HF myocardium.

### 3.2 The Number of Macrophages Is Dramatically Increased in HF and Correlates Strongly With the Accumulation of Neutrophils

**FIGURE 4:**
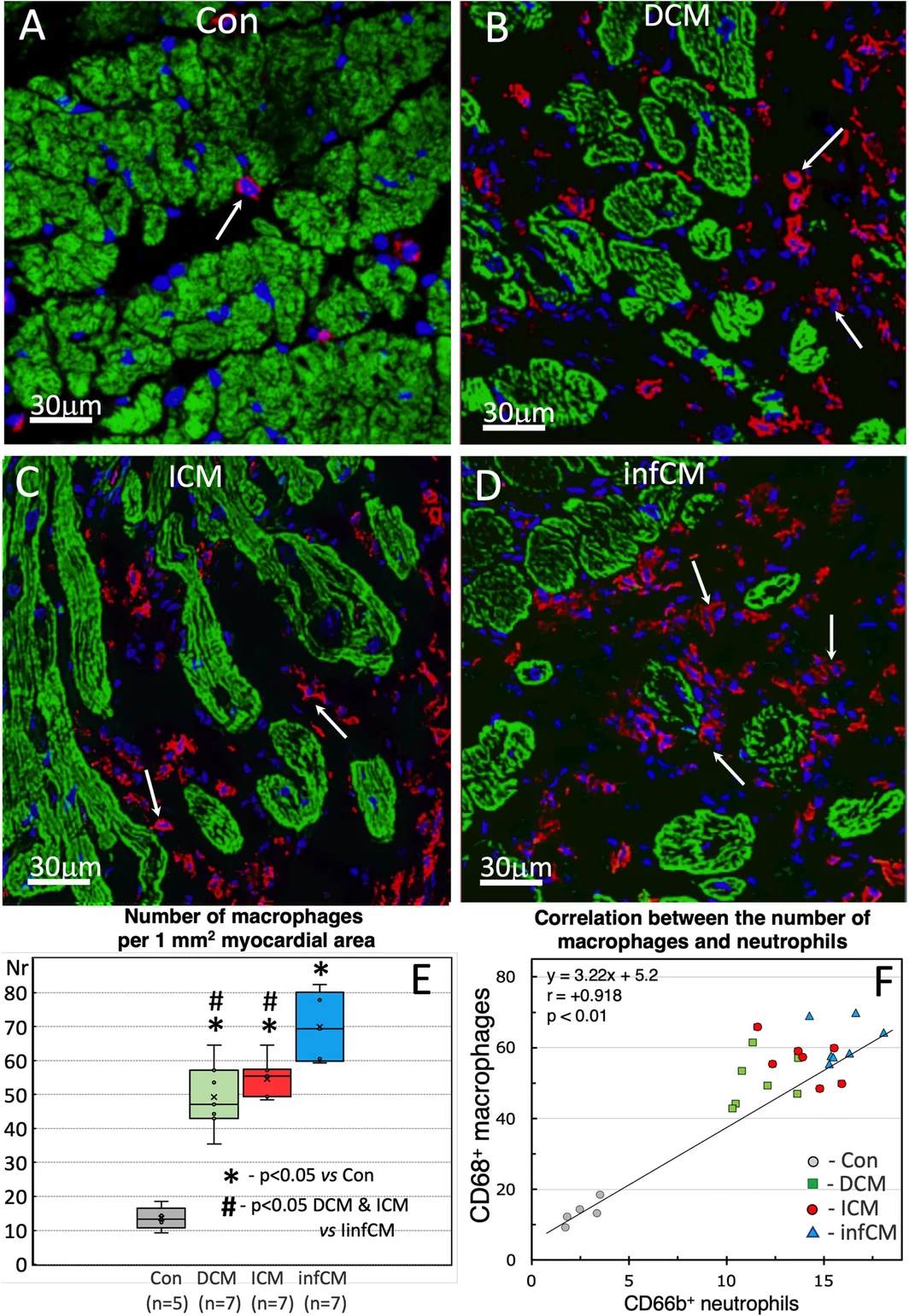
In HF patients, the number of macrophages is increased and correlates positively with the number of neutrophils. Representative immuno‐fluorescence images of CD68‐positive cells (red, arrows) in control groups (A), DCM (B), ICM (C) and infCM (D) as well as the quantitative analysis of macrophages in the examined groups (E). The total number of macrophages per myocardial tissue area correlates significantly with the number of neutrophils (F).

To quantify the number of macrophages, a CD68 antibody as a pan‐macrophage marker was used. Representative micrographs of CD68 labelling in the examined groups are documented in Figure 4A–D. Quantitative analysis revealed that 1 mm^2^ of the myocardial surface in the control group comprised a median of 13.3 [10.8–16.45] macrophages (Figure 4E) whereas the same ventricular myocardial surface comprised a median of 47.1 [42.9–57.1] macrophages in DCM, 55.4 [49.4–57.4] in ICM and 69.4.5 [60.5–79.3] in infCMP (Figure 4E). The difference between DCM and infCM was significantly different (*p* < 0.01). The increase in the number of macrophages indicates the presence of chronic myocardial inflammation in HF patients. Furthermore, the elevation in macrophage count correlated very strongly (*r* = 0.918, *p* < 0.01) with the number of CD66b‐positive neutrophils (Figure 4F).

### 3.3 HF Is Characterised by Macrophage Polarisation Towards the M1‐Like Proinflammatory Phenotype

**FIGURE 5:**
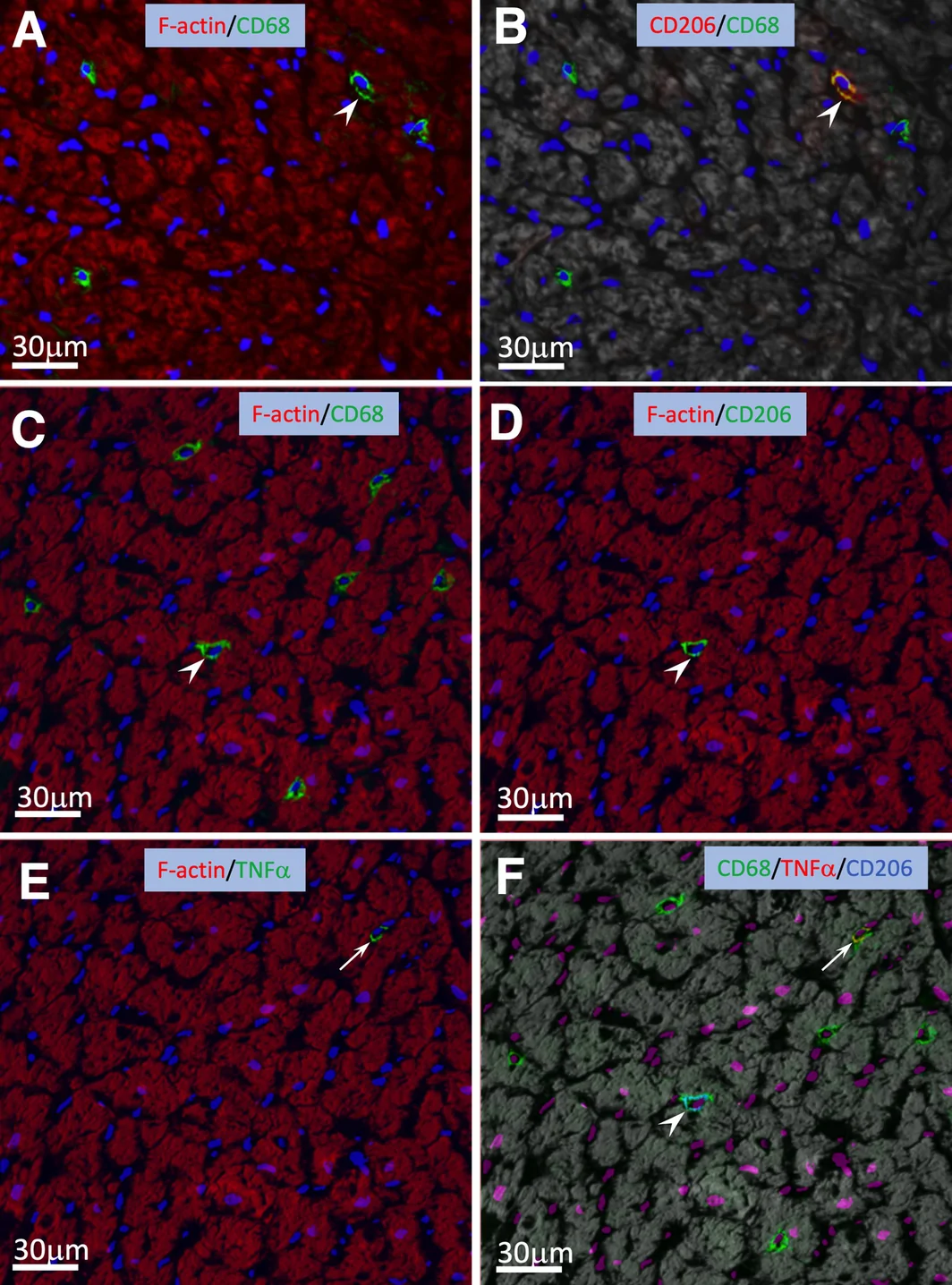
The phenotype of macrophages in the control group. Representative confocal images of double labeling of CD68 (green) and CD206 (arrowhead in B) in a control myocardium (A, B). F‐actin is labelled in red (A) and grey (B). Triple labeling for CD68 (C) with CD206 (arrowhead in E and F) and TNF‐α (arrow in E and F) in a control patient. Note that of seven CD68‐positive cells (C), only one cell is CD206‐positive (D) and another cell is TNF‐α‐positive (E). Panel (F) is the superimposition of all three markers; the cell nuclei are coloured pseudo‐magenta, and F‐actin is shown in grey.

**FIGURE 6:**
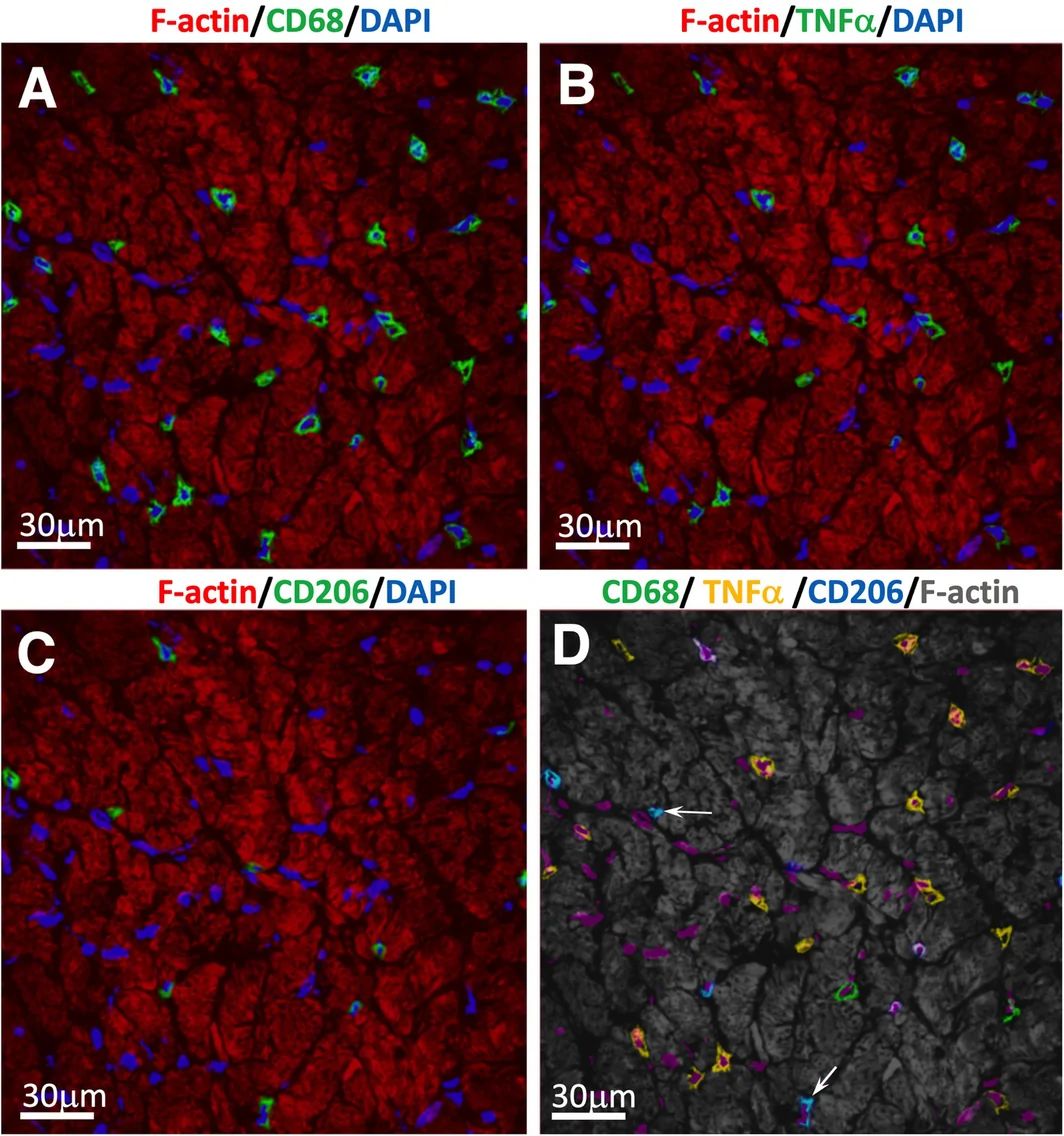
The phenotype of macrophages in a patient with DCM. Representative confocal image of triple labeling for CD68 (A), TNF‐α (B) and CD206 (C). Panel D is the overlay of all three labels and shows that the majority of yellow‐labelled macrophages are TNF‐α‐positive cells that are mixed with some CD206‐positive cells (arrows). In panel (D), the cell nuclei are coloured pseudo‐magenta, and F‐Actin is shown in grey.

**FIGURE 7:**
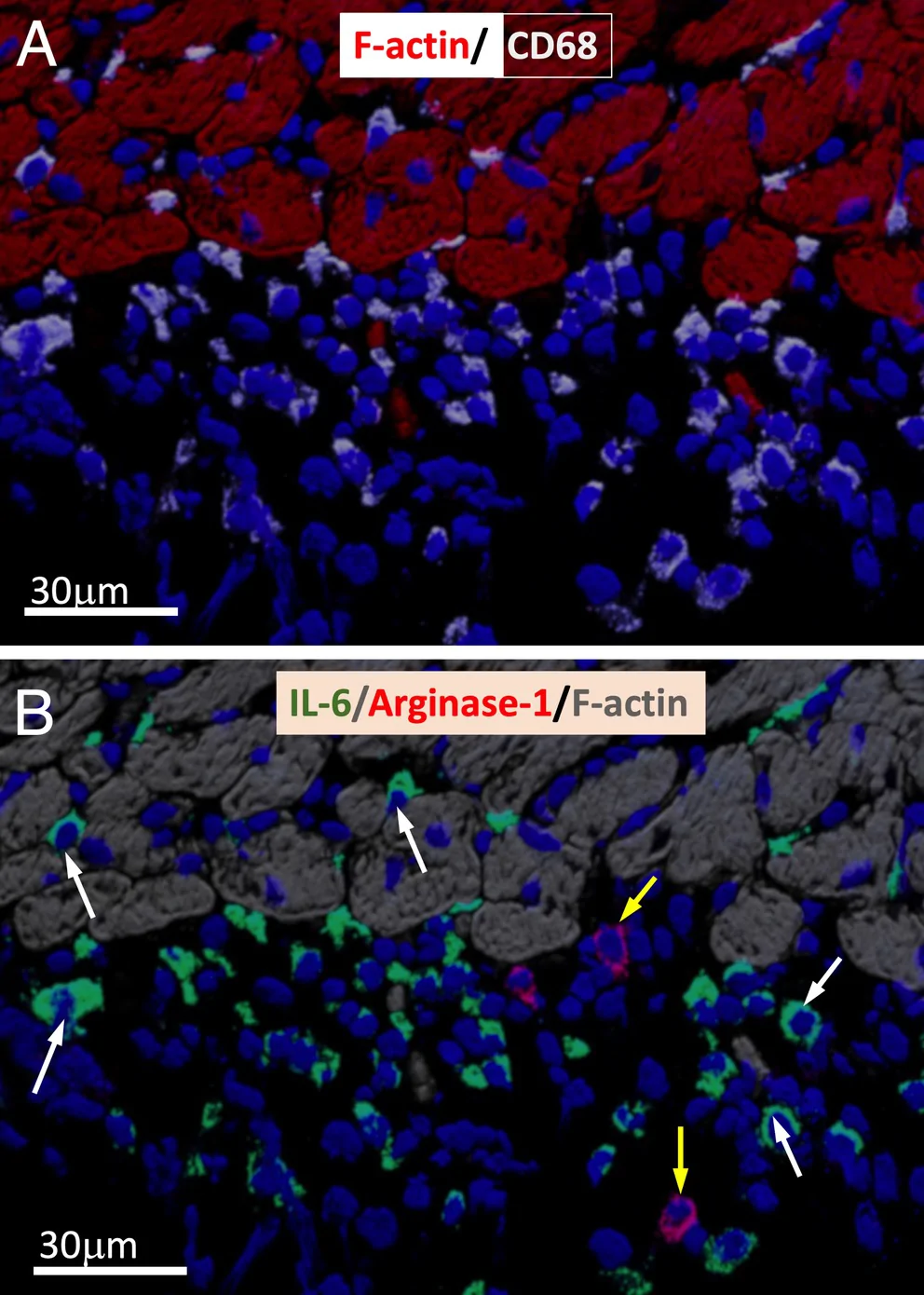
The M1‐like macrophages predominate over M2‐like macrophages in a patient with infCM. Triple labeling of CD68 (grey in A), IL‐6 (green in B) and arginase 1 (red in B). Note that IL‐6‐positive cells (white arrows) are more numerous than arginase 1‐positive cells (yellow arrows). F‐actin is marked red in (A) and grey in (B). Cell nuclei are stained blue with DAPI.

To characterise the phenotype of macrophages in tissue sections, we used markers recommended in the general nomenclature and experimental guidelines of Murray et al. [44]. Specifically, to characterise the M1‐like phenotype we used antibodies against TNF‐α and IL‐6. For identification of M2‐like macrophages we employed anti‐CD206 and arginase‐1 antibodies. Figure 5 shows examples of macrophage characterisation in control tissue, where only a small proportion of CD68‐positive macrophages that were positive for either TNF‐α or CD206 were found. In marked contrast, the HF groups, regardless of their aetiology, not only had an increased number of macrophages, but also showed a prevalence of TNF‐α and IL‐6–positive cells over CD206‐ or arginase‐1‐positive M2‐like macrophages. Typical examples of such a relationship between M1‐like and M2‐like macrophages are documented in samples of patients with DCM (Figure 6) and infCM (Figure 7).

**FIGURE 8:**
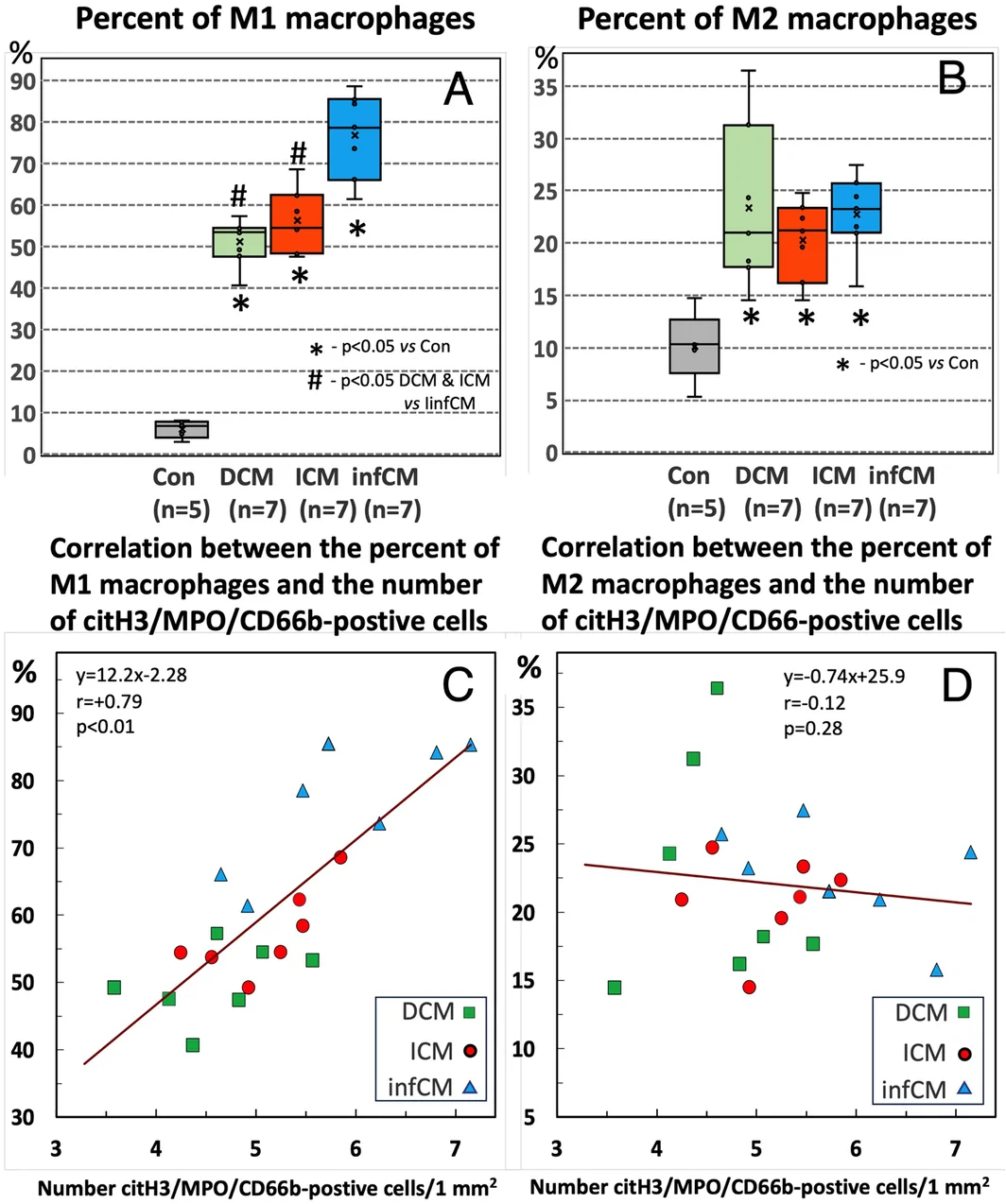
Boxplots of the percentage of CD66b^+^/TNF‐α^+^ M1‐ and CD66b^+^/CD206^+^ M2‐like macrophages in the examined groups (A, B) and the correlation analysis between these cells and the number of citH3/MPO/CD66b‐positive cells (C, D).

Quantitative analysis of the percentage of M1‐like macrophages revealed a value of 78.5% [69.8–84.7] that differed significantly (*p* < 0.05) from the median percentage of M1‐like macrophages in patients with DCM (53.2% [48.4–54.4]) or ICM (54.5% [51.1–60.3]). The median percentage of M1‐like macrophages in the control group was 3% [1.6–5.5] and differed significantly (*p* < 0.01) from all HF groups (Figure 8A).

Quantitative analysis of the percentage of M2‐like macrophages indicated a value of 20.9% [17.9–27.7] in DCM, 21.1% [17.8–22.8] in ICM, and 23.2% [21.2–25.1] in infCM, respectively. The median percentage of M2‐like macrophages in the control group was 6.5% [4.8–7.6] and differed significantly (*p* < 0.01) from all HF groups (Figure 8B).

The significantly higher increase in M1‐like macrophages compared to M2‐like macrophages was also reflected in the M1/M2 ratio (Figure S2). The median of this ratio was 3.19 [2.5–4.3] in patients with infCM and differed significantly (*p* < 0.05) from the median of the M1/M2 ratio in patients with DCM (2.51 [2.2–2.8]) and ICM (2.63 [2.3–2.8]). The median of the M1/M2 ratio in the control group was 0.63 [0.57–0.79] and differed significantly (*p* < 0.01) from all HF groups.

### 3.4 Elevated NETosis in HF Correlates Significantly With High Number of M1‐Like Macrophages

**FIGURE 9:**
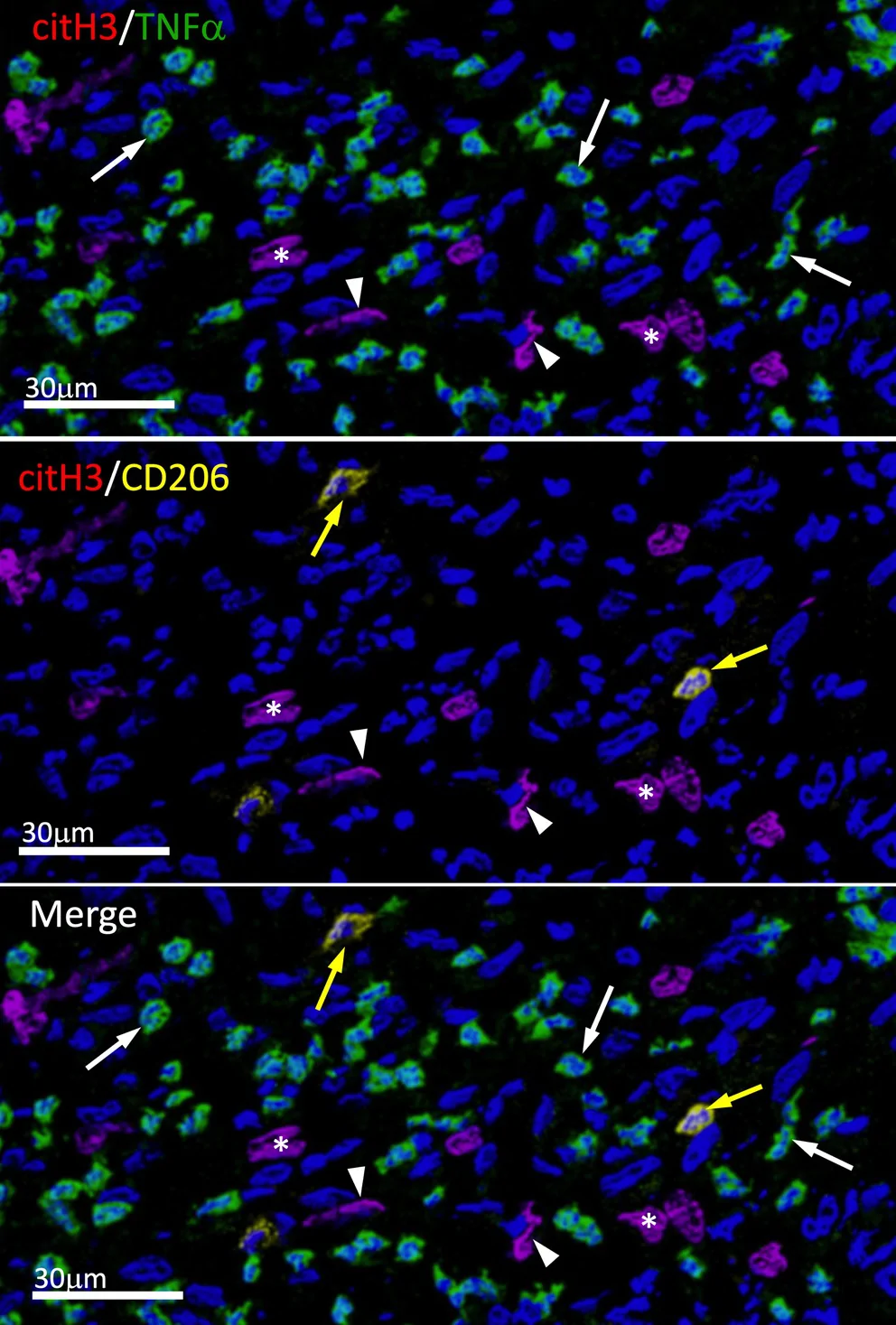
NETosis is more strongly associated with the number of M1‐like rather than with M2‐like macrophages. Representative confocal images of triple labeling for citH3 (purple colour resulting from co‐localization of the red signal of citH3 with the blue colour of DAPI), TNF‐α (white arrows) and CD206 (yellow arrows) in a patient with ICM. Note that citH3 positivity is accompanied by the higher number of TNF‐α‐positive cells rather than with some CD206‐positive cells. Asterisks denote nuclear citH3 and arrowheads indicate typical long NET structures.

When the association between macrophage activation towards a M1‐like phenotype and increased NETosis in the setting of HF was tested, Spearman's correlation analysis revealed a statistically significant positive correlation between the percent of M1‐like macrophages and the number of citH3/MPO/CD66b‐positive (*r* = +0.79, *p* < 0.01, Figure 8C) or citH3/CD66b‐positive neutrophils (*r* = +0.68, *p* < 0.01, Figure S3A). In contrast, there was no significant correlation between the percent of M2‐like macrophages and the number of citH3/MPO/CD66b‐positive neutrophils (*r* = −0.12, *p* = 0.28, Figure 8D) or citH3/CD66b‐positive neutrophils (*r* = −0.18, *p* = 0.31, Figure S3B). Moreover, triple labeling of tissue sections with antibodies against citH3, TNF‐α, and CD206 supported this observation, with results (Figure 9) demonstrating that the citH3‐positive cells are surrounded by many more TNF‐α‐positive than CD206‐positive macrophages.

In summary, these data indicate that, in the settings of HF, there is a shift in macrophage activation towards a proinflammatory M1‐like phenotype in connection with the increased production of NETs.

## 4 Discussion

In this study, we demonstrate that HF is characterised by an increased number of neutrophils, which correlates significantly with an elevated number of macrophages in the myocardium. Furthermore, earlier observations were confirmed in that the number of neutrophils that develop NETosis and the key proteins involved in this process are dramatically increased in the ventricular myocardium of patients with HF, regardless of aetiology [62, 63]. Importantly, we show that increased NETosis in the myocardium of patients with HF is associated with macrophage activation towards a proinflammatory M1‐like phenotype.

The interaction between macrophages and neutrophils that have undergone NETosis has garnered considerable research interest. Recently, it has been suggested that NETs could be removed by macrophages via phagocytosis [52]. It is generally believed that M2‐like macrophages, rather than M1‐like macrophages, primarily participate in the clearance of dead cells [64]. Nakazawa et al. demonstrated in co‐culture models of phorbol myristate acetate‐stimulated neutrophils with monocyte‐derived macrophages that both M1‐ and M2‐like macrophages can degrade NETs in a time‐dependent manner. During the early phase of NET formation, mainly M1‐like macrophages are involved in phagocytosing NET structures, a process completed later by M2‐like macrophages [53]. Yu et al. studied isolated neutrophils and alveolar macrophages from patients or mice with acute pulmonary ischemia/reperfusion and revealed a mutual, vicious feedback loop between NETosis and M1‐like macrophages [55]. In this context, our study, which shows a close interaction between increased NETosis and M1‐like macrophage polarisation, suggests that both processes may interact and together promote and sustain chronic myocardial inflammation, a hallmark of HF. Conversely, since M1‐like macrophages are involved in the phagocytosis of neutrophils undergoing NETosis [53], our observation of heightened M1 polarisation indicates that this could be a compensatory mechanism to protect the myocardium from excessive NETosis, although it appears unable to inhibit NETosis itself. This hypothesis is supported by a further increase in NETosis, which promotes M1‐like macrophage polarisation [51].

An important and perhaps unexpected observation in our study was the increased polarisation of M2‐like macrophages, though to a lesser degree than M1 polarisation. M2‐like macrophages have reparative, profibrotic, and phagocytic properties [26, 29, 31, 37, 41, 46, 65, 66]. Since cardiomyocyte cell death and myocardial fibrosis are important components in the pathogenesis of HF [67, 68, 69], an increase in M2 polarisation suggests they may be involved in the clearance of dead cells and contribute to myocardial fibrosis.

An interesting aspect of the associations observed in our study between elevated NETosis and M1‐like macrophage polarisation is the question of whether these associations are more of a concomitant feature of HF than a causal factor in its development. Although our observations appear to be more to be a side effect of chronic HF than a causal factor in its development and require further investigation, we reported in our earlier studies that neutrophil and macrophage accumulations in patients with compensated left ventricular hypertrophy prior to the development of overt, clinically‐manifested HF [70]. Moreover, we have previously reported that failing human myocardium in the terminal stage loses approximately 20% of its cardiomyocytes each year [67, 71]. Such massive cell death of cardiomyocytes inevitably leads to a reactive inflammatory response involving neutrophils and macrophages. In addition, based on recent research, myocardial extracellular vesicles released by injured cardiomyocytes act as key intercellular messengers that modulate immune responses, including the promotion of neutrophil‐driven inflammation, NETosis and macrophage polarisation [72, 73, 74, 75]. Similar results were reported in other diseases including atherosclerosis, thromboinflammation, hemorrhagic stroke, valvular heart diseases, tumours, and systemic lupus erythematosus [75, 76, 77].

In summary, our findings demonstrate that the pathophysiologic development and progression of HF and its proinflammatory status are associated with dynamic interactions between neutrophils undergoing NETosis and macrophage polarisation. To develop and expand this issue in clinical terms, the inhibition of NETosis [78] and the maintenance of the balance between M‐1 and M2‐like macrophages [50] in HF patients would be next steps to prove the clinical relevance of the present findings.

### 4.1 Study Limitations

The results of the present study should be interpreted in light of certain limitations, since the study groups were characterised by different etiologies of CMP. Another important limitation was the small number of investigated patients resulting in the high interindividual variability of the parameters studied. Nonetheless, our study demonstrated important associations of the elevated NETosis and M1‐like macrophage polarisation to justify our conclusions.

Another important aspect is the reciprocal interaction between NETs and macrophages, which creates a positive feedback loop and thereby exacerbates inflammatory responses and tissue damage [77]. This aspect requires further investigation as to whether chronic HF involves a bidirectional crosstalk between neutrophils, which tend to undergo uncontrolled NETosis, and macrophage polarisation, mirroring thus observations from experimental models of post‐myocardial infarction [8, 79]. The results of this study suggest that there is a delicate balance between neutrophils and macrophages in patients with HF and that understanding this process will be necessary for the development of new therapies for chronic HF.

## 5 Conclusions

Accumulations of neutrophils and macrophages are typical features of chronic myocardial inflammation in HF, regardless of its aetiology. An increased number of neutrophils undergoing NETosis in HF is associated with the activation of myocardial macrophages and switches these cells towards a proinflammatory M1‐like phenotype.

## Author Contributions

**Sawa Kostin:** conceptualization, investigation, writing – original draft, visualization, validation, data curation, supervision, project administration, methodology, writing – review and editing, software. **Manfred Richter:** investigation, writing – original draft, writing – review and editing, formal analysis. **Nikolaos Pagonas:** funding acquisition, writing – original draft, writing – review and editing, formal analysis, supervision. **Florian Krizanic:** writing – original draft, writing – review and editing, formal analysis. **Theodoros Kelesidis:** writing – original draft, writing – review and editing, validation, formal analysis. **Gerasimos Siasos:** writing – original draft, writing – review and editing. **Hector A. Cabrera‐Fuentes:** conceptualization, writing – original draft, writing – review and editing, formal analysis, validation, methodology, investigation. **William A. Boisvert:** writing – original draft, writing – review and editing, formal analysis. **Klaus T. Preissner:** formal analysis, writing – review and editing, writing – original draft. **Oliver Ritter:** writing – original draft, writing – review and editing, formal analysis.

## Funding

The authors have nothing to report.

## Ethics Statement

This study was approved by the ethical committee of the Landesaerztekammer Hessen (project number FF 8/2011 and FF56/201).

## Consent

All authors have approved the contents of this manuscript and provided consent for publication.

## Conflicts of Interest

The authors declare no conflicts of interest.

## Supporting information


**Figure S1:** (A, B) Representative Western blot of citH3 and its quantification in the groups studied. (C) RT‐qPCR of the MPO gene normalised to GADPH in the groups studied. Values are presented as median with IQR.

**Figure S2:** The ratio of M1‐ to M2‐like macrophages in the groups studied. Note that the M1/M2 ratio in patients with infCM is statistically different from that in DCM and ICM patients.

**Figure S3:** Correlation analysis between the number of citH3/CD66b‐positive neutrophils and the percent of CD68TNF‐α‐positive M1 macrophages (A) or CD68/CD206‐positive M2 macrophages (B).

**Table S1:** Clinical baseline characteristics of patients.

## Data Availability

The data that support the findings of this study are available from the corresponding author upon reasonable request.
